# Evaluation of oral health conditions among children with special health care needs using clinical records

**DOI:** 10.3389/fpubh.2026.1738526

**Published:** 2026-04-07

**Authors:** Anran Wang

**Affiliations:** Nanjing Stomatological Hospital, Affiliated Hospital of Medical School, Institute of Stomatology, Nanjing University, Nanjing, China

**Keywords:** children with special health care needs, China, clinical records, dental caries, gingivitis, oral hygiene, plaque, risk factors

## Abstract

**Background:**

Oral health is a vital component of overall well-being, yet children with special health care needs (CSHCN) are disproportionately affected by dental diseases due to physical, cognitive, behavioral, and systemic factors.

**Methods:**

A retrospective cross-sectional analysis was conducted on clinical records of 500 CSHCN aged 2–17 years, collected from pediatric dental clinics and special care centers in Nanjing, China. Lifestyle, nutrition, oral health, and socio-demographic data were extracted. Caries experience (dmft/DMFT), gingival condition, plaque accumulation, malocclusion, and dental hygiene were assessed. Chi-square tests and one-way ANOVA explored associations between clinical diagnosis and oral health outcomes. Multiple logistic regression identified factors independently associated with dental caries.

**Results:**

The results comprised 62% males and 76% urban residents. Neurodevelopmental disorders predominated (ASD 26%, ID 18%), followed by cerebral palsy (17%) and Down Syndrome (8%). Caries prevalence was high (77%), with a mean dmft of 3.46 ± 2.15 and DMFT 2.38 ± 1.80. Gingivitis was observed in 72.6% and poor oral hygiene in 52.2% of children. Malocclusion affected 44% of participants, and only 21.4% had a dental visit in the past year. Oral hygiene practices were suboptimal: 56% brushed once daily, 40% received assisted brushing, and 42% used fluoridated toothpaste. Regression analysis identified poor oral hygiene (AOR = 2.85, *p* < 0.001), high plaque index (AOR = 2.20, *p* = 0.003), gingivitis (AOR = 1.65, *p* = 0.046), brushing <2/day (AOR = 1.92, *p* = 0.021), non-use of fluoridated toothpaste (AOR = 1.75, *p* = 0.026), and frequent sugar intake (AOR = 2.44, *p* = 0.001) as factors significantly associated with dental caries. Age was also associated with increased caries risk, while sex, BMI, residence, and diagnosis category were not.

**Conclusion:**

Special needs children have increased rates of dental cavities, gingivitis, plaque accumulation, and malocclusion, as well as poor oral hygiene and limited preventive care. Dental caries risk is associated with oral hygiene practices, sugar intake, and fluoride use, suggesting the need for targeted preventive measures, caregiver education, and improved CSHCN-specific dental services.

## 1 Introduction

Oral health is a critical component of overall health, influencing nutrition, communication, self-esteem, and quality of life. Special Health Care Needs (CSHCN) children, who have physical, developmental, behavioral, or emotional issues and require health and related services beyond what is expected of children in general, are at risk for poor dental health (1, 2). These children often suffer from oral hygiene issues due to physical or cognitive disabilities, behavioral disorders, or their need for daily care. Certain medicines, diets, and systemic disorders may also increase the risk of dental caries, periodontal disease, and other oral health issues. Oral health issues can cause CSHCN patients’ pain, discomfort, functional limitations, and social shame. Individual health and quality of life can be affected by all of these factors (3, 4). In China, where over 1.4 billion people live, dental health is a major issue, especially for special needs. National surveys show that 50 to 70% of 5–12-year-olds have dental caries, with many untreated. By age 5, caries prevalence in preschoolers aged 3–5 rises to about 72%. In northeast China, 78.6% of visually impaired schoolchildren had caries, and just 11.6% of decaying teeth in the mixed dentition had been repaired. In Guangzhou, 53.5% of 12–17-year-olds with intellectual impairments had dental caries, with a mean DMFT score of 1.5. These data show that CSHCN in China have a far greater rate of oral disorders than their normally developing peers, sometimes without access to restorative or preventive care (5–7).

The causes and risk factors for poor oral health in CSHCN are multifactorial. Physical and cognitive impairments may limit the child’s ability to perform effective oral hygiene, while behavioral challenges can delay cooperation during brushing or dental visits (8). Many children use medications such as anticonvulsants, antipsychotics, or antihistamines, which may reduce salivary flow, alter oral microbial balance, or cause gingival overgrowth, thereby increasing susceptibility to dental caries and periodontal disease (9). Sweet drinks, prolonged bottle feeding, and swallowing issues increase dental caries risk as do pureed foods. Socioeconomic determinants and parental education are equally essential, since poor caregiver knowledge, oral health literacy, and access to expert dental services increase untreated illness rates (10, 11). Cerebral palsy, congenital heart disease, ASD, and Down syndrome can complicate dental care. These aberrations cause enamel flaws, malocclusion, delayed eruption, and cavity susceptibility (12–14). Dental issues can impact diet, speech, social life, and quality of life. Poor oral hygiene can cause infective endocarditis in children with congenital heart disease. Dental neglect in rural and underdeveloped China worsens these issues for disabled children. Need targeted prevention and treatment (15). Pediatric dental research must encourage equitable and effective care that improves oral and general health outcomes for affected children because oral health affects overall well-being (16, 17). Due to the high frequency of oral illness, restricted access to restorative therapy, and complicated multifactorial risk factors in special needs children, a complete oral health evaluation is needed. Thus, this study seeks to identify the most common oral health issues, risk factors, service gaps, and preventive approaches. Clinical records can drive focused treatments, caregiver education, and public health planning in China, where healthcare access varies and special needs data is rare. Improved oral hygiene, treatment access, and complete oral and systemic health care for special needs children depend on this evaluation.

## 2 Materials and methods

### 2.1 Study design and setting

This was a retrospective cross-sectional analysis of special needs children’s clinical data. Data were obtained from pediatric dental clinics, Nanjing, China, between 10th April, 2023, and 15th April, 2025. This research was approved by the Ethics Committee of Nanjing Stomatological Hospital. No. NJSH-2023NL-36, which followed the Declaration of Helsinki. All patient data was anonymized for privacy. Since this was a retrospective examination of existing records, the ethics committee waived informed consent, but all data handling followed institutional and national privacy and data protection requirements.

### 2.2 Study population

The study initially enrolled 610 patients’ records, aged 2–17 years, with physical challenges, developmental delays, intellectual disabilities, sensory impairments, or chronic medical illnesses requiring specialized care. After applying the inclusion and exclusion criteria, 500 patients’ data were analyzed. Children who were within the age range, had one of the disorders, and had comprehensive clinical and dental health records were included. Records were excluded if essential clinical or oral health data were missing.

### 2.3 Oral health assessment

Since this was a retrospective study, all behavioral and oral health data were obtained from standardized case proformas completed during routine dental visits; no new questionnaires were administered. Data were extracted using a structured form and validated clinical indices, with two researchers independently reviewing records and a 10% subset rechecked for reliability (Cohen’s *κ* ≥ 0.80). Oral health data were retrospectively extracted from patient clinical records. Examinations had been previously conducted by trained pediatric dentists following standardized protocols. Dental caries was recorded using the dmft/DMFT indices, and gingival health using the Löe and Silness Gingival Index (18), plaque accumulation using the Silness & Löe Plaque Index, and overall oral hygiene using the Simplified Oral Hygiene Index (OHI-S) (19). Malocclusion was classified according to Angle’s classification, and any history of dental trauma was noted. Behavioral and preventive information, including tooth brushing frequency, caregiver-assisted brushing, and regular dental visits, was also obtained from the clinical records.

### 2.4 Statistical analysis

Data were entered and analyzed using SPSS version 25. Descriptive statistics were calculated for all variables, including frequencies, percentages, means, and standard deviations. The association between oral health outcomes and categorical variables was assessed using chi-square tests. In contrast, continuous variables were compared using ANOVA as appropriate. Logistic regression analysis was performed to identify independent predictors of dental caries and periodontal disease. For regression analyses, *β* = regression coefficient; SE = standard error; AOR = adjusted odds ratio; 95% CI = 95% confidence interval. A *p*-value <0.05 was considered statistically significant.

## 3 Result and discussion

### 3.1 Socio-demographic, lifestyle, and nutritional profile of participants

The study sample comprised 500 children with special health care needs. In terms of age distribution, the largest proportion (30.0%) fell into the 7–9-year age group, followed by 10–12 years (26.0%), 4–6 years (18.0%), 13–17 years (20.0%), and the youngest 2–3 years (6.0%). Male children outnumbered females at 62.0% versus 38.0%. The majority resided in urban areas (76.0%), with 24.0% from rural settings. Socio-economic status was distributed with 50.0% in the middle category, 30.0% in the low, and 20.0% high. Parental education was high: nearly half (48.0%) had a tertiary education, 40.0% secondary, 10.0% primary, and 2.0% no formal education. Regarding parental occupation, 40.0% of caregivers were skilled workers, 36.0% professional/service, and 24.0% unemployed/homemaker. Most families (85.0%) consisted of ≤4 members. Monthly household income in Chinese Yuan (CNY) showed 54.0% earning 5,000–15,000, 30.0% earning >15,000, and 16.0% earning <5,000. Consanguineous marriage was rare (1.0%). School attendance was reported at 76.0%; among these, 57.9% attended regular schools, 26.3% special education centers, and 15.8% home-based learning. The primary caregiver was the mother in 72.0% of cases, the father in 16.0%, and other family members in 12.0%. In terms of diet, 64.0% ate homemade food, 28.0% mixed home + outside, and 8.0% mainly outside food. Snack/sweet consumption was ≤1 time/day in 24.0%, 2–3 times/day in 33.2%, and >3 times/day in 42.8%. Using WHO growth-standards for BMI, 18.0% were underweight (<− 2 SD), 62.0% normal weight (−2 to +1 SD), 12.0% overweight (+1 to +2 SD), and 8.0% obese (> + 2 SD) (Table 1).

**Table 1:** Socio-demographic, lifestyle, and nutritional profile of children with special health care needs.

Variable	Category	*n*	%
Age group (years)	2–3	30	6.0
	4–6	90	18.0
	7–9	150	30.0
	10–12	130	26.0
	13–17	100	20.0
Sex	Male	310	62.0
	Female	190	38.0
Residence	Urban	380	76.0
	Rural	120	24.0
Socio-economic status	Low	150	30.0
	Middle	250	50.0
	High	100	20.0
Parental education	No formal education	10	2.0
	Primary	50	10.0
	Secondary	200	40.0
	Tertiary	240	48.0
Parental occupation	Unemployed/homemaker	120	24.0
	Skilled worker	200	40.0
	Professional/service	180	36.0
Family size	≤ 4 members	425	85.0
	> 4 members	75	15.0
Monthly household income (CNY)	< 5,000	80	16.0
	5,000–15,000	270	54.0
	> 15,000	150	30.0
Consanguineous marriage (parents)	Yes	5	1.0
	No	495	99.0
School-going status	Attending school	380	76.0
	Not attending school	120	24.0
Type of schooling (of school-going, *n* = 380)	Regular school	220	57.9
	Special education center	100	26.3
	Home-based learning	60	15.8
Primary caregiver	Mother	360	72.0
	Father	80	16.0
	Other family member	60	12.0
Diet type	Home-made food	320	64.0
	Mixed (home + outside)	140	28.0
	Mainly outside food	40	8.0
Sweet/snack consumption frequency	≤ 1 time/day	120	24.0
	2–3 times/day	166	33.2
	> 3 times/day	214	42.8
BMI category (WHO growth standards)	Underweight (< −2 SD)	90	18.0
	Normal weight (−2 SD to +1 SD)	310	62.0
	Overweight (+1 SD to +2 SD)	60	12.0
	Obese (> + 2 SD)	40	8.0

Data presented in *n* (%).

### 3.2 Clinical diagnoses of children with special health care needs

The study documented the clinical diagnoses of children with special health care needs to understand their oral health challenges. In our sample of 500 children with special health care needs, the distribution of clinical diagnoses was as follows: 130 children (26.0%) had autism spectrum disorder (ASD), 90 (18.0%) had Intellectual Disability (ID), and 35 (7.0%) had attention-deficit hyperactivity Disorder (ADHD). Among neurological & motor disorders, 85 children (17.0%) had Cerebral Palsy (CP) and 60 (12.0%) had Epilepsy (with or without comorbidity). In the genetic/chromosomal disorders category, 40 children (8.0%) had Down Syndrome, and 20 (4.0%) had other chromosomal/genetic syndromes (e.g., Prader–Willi syndrome, Fragile X syndrome). Within sensory & communication disorders, 20 (5.0%) had hearing impairment, 10 (2.0%) had visual impairment, and 10 (2.0%) had isolated speech or language delay (Table 2).

**Table 2:** Clinical diagnoses among children with special health care needs.

Clinical diagnosis/condition	*n*	%
Neurodevelopmental disorders		
Autism spectrum disorder (ASD)	130	26.0
Intellectual disability (ID)	90	18.0
Attention deficit hyperactivity disorder (ADHD)	35	7.0
Neurological & motor disorders		
Cerebral palsy (CP)	85	17.0
Epilepsy (with or without comorbidity)	60	12.0
Genetic & chromosomal disorders		
Down syndrome	40	8.0
Other chromosomal/genetic syndromes (e.g., Prader–Willi, Fragile X)	25	5.0
Sensory & communication disorders		
Hearing impairment	15	3.0
Visual impairment	10	2.0
Speech or language delay (isolated)	10	2.0
Total	500	100.0

Data presented in *n* (%).

### 3.3 Oral health status of children with special health care needs

The oral health status of children with special health care needs was assessed to evaluate hygiene and dental care needs. The oral health assessment of 500 children with special health care needs revealed a high burden of dental disease. Caries experience was observed in 385 children (77.0%), while 115 children (23.0%) were caries-free. The mean dmft score for deciduous teeth was 3.46 ± 2.15, and the mean DMFT for permanent teeth was 2.38 ± 1.80. Based on dmft/DMFT indices, 42.0% of children had mild caries (1–3 teeth affected), 25.0% had moderate caries (4–6 teeth), and 10.0% were classified as having severe caries (≥7 teeth affected). According to the Löe and Silness Index, 18.0% of children had healthy gingiva, 40.0% had mild, 22.6% moderate, and 10.0% severe gingivitis. According to the Silness & Löe index, 13.2% of children were fair, 69.8% bad, and 17.0% good due to plaque formation. According to OHI-S, 22.0% of children had good oral hygiene, 25.8% fair, and 52.2% bad. Only 9.0% of youngsters had dental trauma. 56.0% of children had Angle’s Class I occlusion, 28.0% Class II, and 16.0% Class III. 56.0% of youngsters brushed every day, 26.0% twice daily, and 18.0% seldom. Only 40% of children brushed their teeth with assistance, whereas 60% did so alone. Only 21.4% of children had a dental appointment in the past year, indicating insufficient preventive care (Table 3).

**Table 3:** Oral health status among children with special health care needs.

Oral health parameter	Category/range	*n*	%
Caries experience (any tooth affected)	Present	385	77.0
	Absent	115	23.0
Mean dmft (deciduous teeth)	Mean ± SD	3.46 ± 2.15	
Mean DMFT (permanent teeth)	Mean ± SD	2.38 ± 1.80	
Caries severity (based on dmft/DMFT index)	Mild (1–3)	210	42.0
	Moderate (4–6)	125	25.0
	Severe (≥7)	50	10.0
	Caries-free	115	23.0
Gingival condition (Löe and Silness Index)	Healthy (0)	90	18.0
	Mild gingivitis (0.1–1.0)	200	40.0
	Moderate gingivitis (1.1–2.0)	113	22.6
	Severe gingivitis (>2.0)	50	10.0
Plaque index (Silness & Löe)	Good (0–0.9)	85	17.0
	Fair (1.0–1.9)	66	13.2
	Poor (≥2.0)	349	69.8
Oral hygiene status (OHI-S)	Good	110	22.0
	Fair	129	25.8
	Poor	261	52.2
Dental trauma (any tooth)	Present	45	9.0
	Absent	455	91.0
Malocclusion (Angle’s classification)	Class I	280	56.0
	Class II	140	28.0
	Class III	80	16.0
Tooth brushing frequency	Once daily	280	56.0
	Twice daily	130	26.0
	Irregular / rarely	90	18.0
Assisted tooth brushing	Yes (by parent/caregiver)	200	40.0
	No (self-brushing only)	300	60.0
Regular dental visit (past 12 months)	Yes	107	21.4
	No	393	78.6

Data presented in *n* (%).

### 3.4 Oral health behaviors and care practices of children with special health care needs

Special needs children struggle with oral hygiene and dental care. Special needs kids have poor oral health habits, according to the assessment. Some 26.0% of youngsters brushed twice a day, whereas most (56.0%) brushed once, and 18.0% barely brushed. 40% had a parent or caregiver help them brush, while 60% did it themselves. Brushing lasted less than 1 min in 36.0% of children, 1–2 min in 46.0%, and more than 2 min in 18.0%. Toothbrush replacement practices varied: 28.0% replaced toothbrushes every ≤3 months, 52.0% every 4–6 months, and 20.0% replaced them irregularly or less frequently. Fluoride usage was limited: 42.0% of children used fluoridated toothpaste, 32.0% used non-fluoridated toothpaste, and 26.0% were unsure or did not use toothpaste. Additional fluoride exposure, such as mouth rinse or professional fluoride varnish, was reported in only 15.0% of children. Dietary behaviors also indicated potential risk for caries, with 33.2% consuming sugary snacks 2–3 times daily, 42.8% more than 3 times daily, and 42.0% consuming sweetened beverages between meals. Home-prepared diets were most common (64.0%), while 28.0% had mixed diets and 8.0% relied mainly on outside food. Feeding practices among younger or dependent children showed that 90.0% had normal oral feeding, whereas 10.0% required tube or assisted feeding. Use of dental floss or other interdental aids was minimal, with only 12.0% using them regularly. Parental supervision during brushing occurred always in 36.0%, sometimes in 44.0%, and never in 20.0% of cases. Dental care utilization was limited: only 21.4% of children visited a dentist in the past 12 months, with 11.4% attending for preventive visits and 10.0% for problem-oriented care. Toothache or caries (49%) was the most common reason for these visits, followed by normal check-ups (33%) and trauma or orthodontic care (25%) (Table 4).

**Table 4:** Oral health behaviors and care practices among children with special health care needs.

Variable	Category	*n*	%
Tooth brushing frequency	Twice daily	130	26.0
	Once daily	280	56.0
	Irregular/rarely	90	18.0
Brushing assistance	Independent brushing	300	60.0
	Assisted by parent/caregiver	200	40.0
Brushing duration	< 1 min	180	36.0
	1–2 min	230	46.0
	> 2 min	90	18.0
Toothbrush replacement frequency	Every ≤ 3 months	140	28.0
	Every 4–6 months	260	52.0
	> 6 months/irregular	100	20.0
Type of toothpaste used	Fluoridated	210	42.0
	Non-fluoridated	160	32.0
	Unsure/no toothpaste	130	26.0
Fluoride exposure (additional sources)	Mouth rinse/fluoride varnish	75	15.0
	None	425	85.0
Sugar intake (snacks/sweets)	≤ 1 time per day	120	24.0
	2–3 times per day	166	33.2
	> 3 times per day	214	42.8
Drinks between meals	Mainly water	290	58.0
	Sweetened beverages	210	42.0
Diet source	Home-prepared food	320	64.0
	Mixed (home + outside)	140	28.0
	Mostly outside food	40	8.0
Feeding type (for younger/dependent children)	Normal oral feeding	450	90.0
	Tube/assisted feeding	50	10.0
Use of dental floss/interdental aids	Regular use	60	12.0
	Occasional/none	440	88.0
Parental supervision during brushing	Always	180	36.0
	Sometimes	220	44.0
	Never	100	20.0
Dental visit in the past 12 months	Preventive visit	57	11.4
	Problem-oriented visit	50	10.0
	No visit	393	78.6
Reason for dental visit	Toothache/caries	45	49
	Routine check-up	32	33
	Other (trauma, orthodontic, etc.)	30	25

Data presented in *n* (%).

### 3.5 Association between clinical diagnosis and oral health variables

Different clinical conditions had different associations between disability and oral health outcomes. ASD (84.6%) and Intellectual Disability (82.2%) had the highest prevalence of caries, followed by Down Syndrome (75.0%), Cerebral Palsy (CP, 72.9%), Epilepsy (73.3%), other genetic or sensory disorders (71.7%), and ADHD (62.9%). Average dmft/DMFT scores were highest in ASD (6.1 ± 2.5) and ID (5.8 ± 2.3), indicating a higher dental decay burden, while ADHD had the lowest score (3.9 ± 1.9). Overall, 82% of children had gingivitis, with Down Syndrome (85.0%) and CP (82.4%) having high rates. In 52.2% of children, Down Syndrome (75.0%) and CP (64.7%) had poor oral hygiene. In 69.8% of children, Down Syndrome (90.0%) and CP (82.4%) had high plaque index scores. ADHD and Down Syndrome had higher rates of high sugar intake (>3 times/day) at 57.1 and 45.0%, respectively. Overall, 42.0% used fluoridated toothpaste, 30.8% in ASD, and 71.4% in ADHD. Dental appointments were limited (21.4% overall), with CP (17.6%) and ADHD (34.3%) children attending the least and most, respectively (Table 5). A substantial correlation (*p* < 0.05) was found between clinical diagnosis and oral health outcomes, including caries prevalence, DMFT/DMFT, gingivitis, poor oral hygiene, and plaque accumulation.

**Table 5:** Association between clinical diagnosis and oral health variables among children with special health care needs.

Clinical diagnosis	Caries present *n* (%)	Mean # #dmft/DMFT ± SD	Gingivitis present *n* (%)	Poor oral hygiene *n* (%)	High plaque index *n* (%)	Sugar intake >3×/day *n* (%)	Fluoridated toothpaste use *n* (%)	Dental visit in last 12 months n (%)	*p*-value
Autism spectrum disorder (*n* = 130)	110 (84.6)	6.1 ± 2.5	95 (73.1)	60 (46.2)	85 (65.4)	45 (34.6)	40 (30.8)	25 (19.2)	0.038*
Intellectual disability (*n* = 90)	74 (82.2)	5.8 ± 2.3	70 (77.8)	50 (55.6)	68 (75.6)	42 (46.7)	35 (38.9)	20 (22.2)	0.028*
Cerebral palsy (*n* = 85)	62 (72.9)	4.7 ± 2.1	70 (82.4)	55 (64.7)	70 (82.4)	38 (44.7)	30 (35.3)	15 (17.6)	0.017*
Epilepsy (*n* = 60)	44 (73.3)	4.9 ± 2.0	42 (70.0)	32 (53.3)	40 (66.7)	26 (43.3)	28 (46.7)	12 (20.0)	0.051
Down syndrome (*n* = 40)	30 (75.0)	5.2 ± 2.4	34 (85.0)	30 (75.0)	36 (90.0)	18 (45.0)	22 (55.0)	10 (25.0)	0.012*
ADHD (*n* = 35)	22 (62.9)	3.9 ± 1.9	18 (51.4)	10 (28.6)	15 (42.9)	20 (57.1)	25 (71.4)	12 (34.3)	0.064
Other genetic/sensory disorders (*n* = 60)	43 (71.7)	4.6 ± 1.9	40 (66.7)	32 (53.3)	35 (58.3)	25 (41.7)	30 (50.0)	13 (21.7)	0.056
Total (*n* = 500)	385 (77.0)	5.2 ± 2.3	363 (72.6)	261 (52.2)	349 (69.8)	214 (42.8)	210 (42.0)	107 (21.4)	—

Data are presented as *n* (%) for categorical variables and mean ± standard deviation (SD) for #continuous variables. Associations between clinical diagnosis and categorical oral health variables were assessed using the Chi-square test, while comparisons of mean dmft/DMFT scores across diagnostic groups were performed using one-way ANOVA. ^*^p < 0.05 indicates statistically significant differences.

### 3.6 Factors associated with dental caries among children with special health care needs

Multiple logistic regression analysis identified several significant predictors of dental caries among children with special health care needs. Age was positively associated with caries risk, with each additional year increasing the odds by 9% (AOR = 1.09; 95% CI: 1.03–1.16; *p* = 0.004). Sex was not significantly associated with caries (AOR = 1.15; 95% CI: 0.78–1.70; *p* = 0.478). Although the diagnosis category showed some variation in caries risk, none of the diagnostic subgroups (ID, CP, Down Syndrome, ADHD/Others) were significantly different from the reference group (ASD) in this model. Oral hygiene status emerged as the strongest predictor: children with poor oral hygiene had nearly three times higher odds of having caries compared to those with good oral hygiene (AOR = 2.85; 95% CI: 1.68–4.85; *p* < 0.001). High plaque index (≥2.0) was associated with over twofold increased odds of caries (AOR = 2.20; 95% CI: 1.30–3.72; *p* = 0.003), and the presence of gingivitis increased the odds by 65% (AOR = 1.65; 95% CI: 1.01–2.70; *p* = 0.046). Tooth brushing less than twice daily nearly doubled the risk of caries (AOR = 1.92; 95% CI: 1.10–3.34; *p* = 0.021), and non-use of fluoridated toothpaste was associated with 1.75 times higher odds (95% CI: 1.07–2.87; *p* = 0.026). High sugar intake (>3 times/day) was another significant predictor, with children consuming frequent sugary snacks having 2.44 times higher odds of caries (95% CI: 1.45–4.09; *p* = 0.001) (Table 6).

**Table 6:** Multiple logistic regression analysis of factors associated with dental caries among children with special health care needs.

Predictor variable	Category/reference	*β* coefficient	SE	Adjusted odds ratio (AOR)	95% CI for AOR	*p*-value
Age (years)	Continuous	0.086	0.030	1.09	1.03–1.16	0.004**
Sex	Male (vs. female)	0.14	0.20	1.15	0.78–1.70	0.478
Diagnosis category	Autism spectrum disorder	—	—	1.00 (ref)	—	—
	Intellectual disability	0.28	0.25	1.32	0.81–2.15	0.260
	Cerebral palsy	−0.16	0.24	0.85	0.52–1.40	0.521
	Down syndrome	0.39	0.31	1.48	0.81–2.71	0.202
	ADHD/others	−0.51	0.31	0.60	0.33–1.11	0.104
Oral hygiene status (OHI-S)	Poor vs. good	1.05	0.27	2.85	1.68–4.85	<0.001***
Plaque Index	High (≥2.0) vs. low (<2.0)	0.79	0.27	2.20	1.30–3.72	0.003**
Gingivitis	Present vs. absent	0.50	0.25	1.65	1.01–2.70	0.046*
Tooth brushing frequency	<2/day vs. ≥2/day	0.65	0.28	1.92	1.10–3.34	0.021*
Fluoridated toothpaste use	No vs. Yes	0.56	0.25	1.75	1.07–2.87	0.026*
Sugar intake frequency	>3/day vs. ≤3/day	0.89	0.27	2.44	1.45–4.09	0.001**
BMI category	Overweight/obese vs. normal	0.21	0.25	1.24	0.75–2.05	0.398
Residence	Rural vs. urban	0.33	0.25	1.39	0.85–2.29	0.187

Data are presented as *β* coefficients, SE, adjusted odds ratios (AOR), and 95% CI for AOR. The table lists categorical variable reference categories. The unit increment model models continuous variables (age). Statistical significance is indicated by *p* < 0.05, with higher levels at *p* < 0.01 and *p < 0.001.

## 4 Discussion

This study assessed the oral health condition of children who have special health care needs through a detailed evaluation. The present study included 500 children with unique health care needs, mostly boys (62.0%) aged 7–9 (30.0%). When compared with previous studies in China and among children with special needs. A Chinese study of disabled children reported a caries prevalence of 63.0% (mean caries 2.77) and found poorer oral health among younger age (<12 yrs) groups (6). In our sample, the majority are urban and middle to high socio-economic status, which may reflect recruitment from urban centers and possibly under-represent children in rural or low-income settings. Another study on 12-year-old adolescents in Shenzhen found dental caries prevalence of 51.2% and noted that lower household registration (non-Hukou) and lower brushing frequency were adverse factors (20). Thus, our demographic profile (with 76% urban residence, relatively high parental education, and a majority middle income) suggests that this cohort may belong to a somewhat advantaged subgroup of children with special needs compared to more deprived populations reported in the literature. On lifestyle and nutritional aspects, our finding that 8% of children mainly consumed outside food and 42.8% snacked >3 times/day resonates with evidence that frequent snacking elevates caries risk in children, including those with special needs. For instance, the preschool-COPSCHN work in China observed associations between sugary consumption and caries (21). Although direct comparison data are limited, the relatively high rate of normal weight (62%) and moderate overweight/obesity (20% combined) indicates that nutritional risk factors in this group may mirror transitions seen in general pediatric populations in China. Overall, the study shows that participants are primarily urban, have moderate to high parental education and wealth, and have mostly normal nutritional status, which may contrast with more disadvantaged groups described elsewhere. Higher socioeconomic level and parental education can safeguard oral health outcomes; however, children with special needs are still vulnerable due to disability-related variables. When comparing with prior studies, for instance, the study among children with ASD found that children with ASD had notably worse oral health status compared with typically developing children in terms of hygiene, access to care, and oral symptoms (22, 23). Another Chinese cross-sectional study of children with intellectual disabilities (aged 12–17 in Guangzhou) reported a caries prevalence of 53.5% and found that the presence of cerebral palsy increased the risk of caries in that group (6). Further, broader reviews report that among children with special needs in Asia (including China), disability types such as ASD, ID, CP, and Down Syndrome are prominent in oral health research (24). Thus, our distribution (ASD 26.0%, ID 18.0%, CP 17.0%, etc.) aligns with the range of disability types typically included in Chinese special-needs oral health research. The relatively high share of ASD and CP in our study is consistent with the literature emphasizing that children with neurodevelopmental and motor disabilities often appear in special-needs dental research due to higher oral health risk and care complexity (25, 26). In summary, the findings reinforce that children with special health care needs in China represent a heterogeneous group of diagnoses (neurodevelopmental, motor, genetic, sensory), with ASD, ID, CP, and Down Syndrome forming the major sub-groups, consistent with previous research. The implication is that interventions and oral health services need to be tailored not only by disability-status broadly but by the specific diagnostic category, since risk profiles and care needs vary by type of condition.

The current study showed that 77.0% of children, caries was present with mean DMFT scores of 3.46 ± 2.15 and 2.38 ± 1.80, with 42.0% having mild, 25.0% moderate, and 10.0% severe caries. The findings match earlier studies showing a higher oral disease burden in special needs children. In India, 53.5% of children with intellectual impairments had caries with a mean DMFT of 1.5 (27), suggesting that neurodevelopmental and motor impairments may increase caries rates, similar to our 77.0% caries prevalence. In northeast China, 78.6% of visually challenged children had caries and poor restorative care (28). It matches our study’s high untreated caries and poor dental visits. Our study’s 72.6% gingivitis rate matches previous reports among CSHCN in China and other Asian populations, where plaque accumulation and gingival inflammation are common due to limited manual dexterity and reliance on caregivers for oral hygiene (29, 30). 44.0% Class II and III malocclusion rate is consistent with past Chinese studies showing that children with cerebral palsy, Down Syndrome, and other genetic disorders are more likely to have developmental dental deformities. Our study indicated that 26.0% brushed twice daily, 40.0% used assisted brushing, and 78.6% had no dental appointment in the past year. The present study findings support previous Chinese research showing that special needs children in moderate socioeconomic metropolitan areas lack preventative treatment and rely on caretakers for oral hygiene (5). CSHCN in India, Saudi Arabia, and Europe have high caries rates, poor gingival health, and low dental visits, demonstrating this is a global concern in pediatric special care dentistry (31–33). Our study found significant rates of dental cavities, gingivitis, plaque accumulation, and malocclusion in youngsters, as well as inadequate oral hygiene and preventative dental care. These data suggest that Chinese special needs children need targeted oral health interventions, caregiver education, and better dental services. These findings support studies demonstrating that special needs children have poor oral hygiene and dental care. Fewer than 30% of Saudi cerebral palsy youngsters brushed twice a day, and 15% received professional dental care. Caretakers also brushed kids (34). Due to caregiver ignorance and lack of professional treatment, Chinese children with intellectual impairments and autism had low fluoride use and few preventative dental checkups (35–37). Despite urban location and moderate socioeconomic status, Chinese children with special health care needs had poor oral health and insufficient preventative dental care, which may increase their risk of caries and gingival disease. Caregiver education, comprehensive oral hygiene, and CSHCN-specific preventative dentistry are needed.

Caries prevalence and severity were highest in ASD and Intellectual Disability children, while ADHD children had the lowest rates and scores. These findings support earlier research showing that children with neurodevelopmental and genetic problems have more oral illness. An ASD study found higher caries, plaque, and gingivitis rates than typically developing youngsters (38), compatible with our findings that ASD had the greatest caries and plaque levels. Research on children with Down Syndrome and cerebral palsy found higher caries risk, poor oral hygiene, and elevated plaque indices related to motor impairments, self-brushing difficulties, and difficulty getting preventive dental care (39, 40). International research also shows that children with ID, ASD, CP, or genetic disorders had higher dental caries, gingival inflammation, and lower preventive treatment rates than neurotypical children (26, 27). Conversely, children with ADHD have lower caries prevalence and oral hygiene deficiencies, which is consistent with a Chinese study that found hyperactive children without major motor or cognitive impairments have better independent oral hygiene (41). This current study found that disability type strongly affects special needs children’s dental health. Children with ASD, ID, CP, and Down Syndrome had the most caries, gingivitis, plaque, and poor dental hygiene. Moderate sensory and behavioral issues. To reduce oral health inequities in this vulnerable population, targeted prevention interventions, caregiver-assisted oral hygiene, and diagnostic-category-specific dental visits are needed. In the multivariable model, BMI, sex, residency, and diagnosis category did not independently predict caries. In earlier investigations of children with exceptional difficulties, high plaque accumulation, infrequent brushing, and excessive sugar intake were consistently linked to increased caries prevalence (42–44). In Pakistan and Saudi Arabia, inadequate caregiver-assisted brushing, low fluoride exposure, and frequent snacking predict dental cavities in CSHCN (45, 46). Risk factor: age matches longitudinal studies showing caries builds with time, especially in children with motor or cognitive deficits (11). Poor brushing, restricted fluoride usage, high sugar intake, and plaque formation promote dental caries in children with specific health care needs, underlining the need for caregiver-assisted oral hygiene, dietary counseling, and preventative measures.

### 4.1 Limitations

Retrospective record limitations and potential caregiver recall bias exist, but the study still provides valuable insights into the dental health and treatment needs of children with special needs. This study used data from clinics in a single urban city, leading to under-representation of rural or low-access CSHCN. Differences in access and referral patterns may influence prevalence estimates, limiting generalizability. Broader, multi-center studies are needed.

## 5 Conclusion

Children with special health care requirements (CSHCN) have significant rates of dental caries, gingivitis, plaque accumulation, and malocclusion, as well as poor oral hygiene and insufficient preventative dental care. The biggest oral disease burden was in neurodevelopmental and motor impairments such as Autism Spectrum Disorder, Intellectual Disability, Cerebral Palsy, and Down Syndrome. Poor oral hygiene, high plaque accumulation, infrequent brushing, non-use of fluoride toothpaste, and frequent sugar consumption were the biggest predictors of dental caries, along with age, according to multiple logistic regression analysis. These findings emphasize the need for focused interventions such as caregiver-assisted oral hygiene programs, dietary counseling, fluoride use promotion, and better children’s dental services. CSHCN oral health disparities can be reduced by tailoring preventive and treatment methods to diagnostic groups. To improve oral health and quality of life for special needs children, oral health management must be integrated into comprehensive care programs.

## Data Availability

The original contributions presented in the study are included in the article/supplementary material, further inquiries can be directed to the corresponding author.
